# Solvent-Free Preparation of 1,8-Dioxo-Octahydroxanthenes Employing Iron Oxide Nanomaterials

**DOI:** 10.3390/ma12152386

**Published:** 2019-07-26

**Authors:** Fatemeh Rajabi, Mohammad Abdollahi, Elham Sadat Diarjani, Mikhail G. Osmolowsky, Olga M. Osmolovskaya, Paulette Gómez-López, Alain R. Puente-Santiago, Rafael Luque

**Affiliations:** 1 Department of Science, Payame Noor University, Tehran 19569, Iran; 2 Department of Chemistry, University of Guilan, Rasht 1914, Iran; 3 Institute of Chemistry, Saint-Petersburg State University, Saint-Petersburg 198504, Russia; 4 Department of Organic Chemistry, University of Cordoba, Campus de Rabanales, Edificio Marie Curie (C-3), Ctra Nnal IV-A, Km 396, E14014 Cordoba, Spain; 5 Peoples Friendship University of Russia (RUDN University), 6 Miklukho Maklaya str., 117198 Moscow, Russia

**Keywords:** iron oxide nanoparticles, xanthenes, multicomponent reactions

## Abstract

In this study, 1,8-dioxo-octahydroxanthenes were prepared employing a simple, effective and environmentally sound approach utilizing an iron oxide nanocatalyst under solventless conditions. The proposed iron oxide nanomaterial exhibited high product yields, short reaction times and a facile work-up procedure. The synthesized catalyst was also found to be highly stable and reusable under the investigated conditions (up to twelve consecutive cycles) without any significant loss in its catalytic activity.

## 1. Introduction

All the natural reactions have at least one catalyst to improve its performance. Nowadays, catalysis is considered as a fundamental pillar in chemistry. Due to the needs of selecting environmentally friendly catalysts to reduce cost issues of the chemical industry [1], the selection of green catalysts has become a key challenge in modern society. Nanocatalysis is an emerging field in catalytic organic transformations. A number of chemical reactions employ nanocatalytic systems due to the larger surface area of nanoparticles compared to their bulk counterparts, giving rise to numerous catalytically active sites which lead the chemical transformations of the adsorbed reactive molecules. For these reasons nanoparticles are considered as suitable heterogeneous catalysts for a wide range of reaction.

Xanthene’s heterocycles and derivatives constitute a relevant type of natural products, featuring relevant biological activities including anti-depressants and antimalarial agents [2], anti-inflammatory [3], antiviral [4], antibacterial [5], and photosensitizers in photodynamic therapy [6]. Xanthene derivatives have also shown interesting properties for fluorescent materials [7], pigments and cosmetics [8] and have been used in biodegradable agrochemicals [9,10] and laser technologies [11].

In recent years, several strategies were disclosed for xanthenes and derivatives syntheses such as intra-molecular phenyl–carbonyl coupling reactions [12], trapping of benzynes by phenols [13], cycloacylation reaction of carbamates [14], cyclodehydrations [15], reaction of aryloxymagnesium halides with triethyl orthoformate [16], reaction of *β*-naphthol with 2-naphthol-1-methanol [17], carbon monoxide [18] and formamide [19].

Xanthene synthesis is catalyzed by many alternative catalysts, such as*p*-dodecylbenzenesulfonic acid [20], NaHSO_4_-SiO_2_ [21], silica sulfuric acid [22], amberlyst-15 [23], InCl_3_/ionic liquid [24], triethylbenzyl ammonium chloride [25], phosphomolybdic acid supported on silica gel [26], HClO_4_-SiO_2_ [27], ZnO and ZnO-acetyl chloride [28], solventless Dowex-50W ion exchange resin protocols [29], SbCl_3_/SiO_2_ [30], silica-supported H_14_[NaP_5_W_30_O_110_] nanoparticles [31], SiO_2_–R–SO_3_H [32], H_3_PW_12_O_40_ supported MCM-41 [33], DABCO–bromine [34], cyanuric chloride [35], TMSCl [36], ZrO(OTf)_2_ [37] and [Et_3_N–SO_3_H]Cl [38]. Other methods have also been documented for such syntheses [39,40,41,42,43], which have disadvantages including the utilization of toxic and/or costly reagents/catalysts/organic solvents, prolonged times of reaction, formation of undesirable or toxic by-products, lack of thermal stability of the reagents and low yields. To overcome the mentioned drawbacks and the growing environmental issues, more effective, practical and benign protocols for xanthenes synthesis and their derivatives represent a promising strategy.

**Scheme 1:**
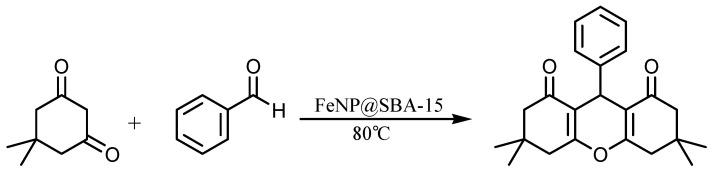
Schematic illustration of the solventless multicomponent synthesis of xantheses catalyzed by FeNP@SBA-15.

Herein, we report on an evaluation of the catalytic activity of an iron oxide nanomaterial based on SBA-15 (FeNP@SBA-15) as active, stable and recyclable heterogeneous catalysts for the preparation of 1,8-dioxooctahydroxanthene and substituted compounds via solventless reaction between aromatic aldehydes and dimedone (Scheme 1).

## 2. Materials and Methods

### 2.1. Synthesis of Iron Oxide Nanocatalyst

A suspension of aminopropyl-functionalized SBA-15 materials (2.35 g, NH_2_ loading 0.85 mmol g^−1^) in an excess of absolute MeOH was combined with Salicylaldehyde (2 mmol, 0.244 g). The mixture color became yellow by imine formation in 6 h, after which Fe(NO)_3_·9H_2_O, (1 mmol) was added. The resulting mixture was slightly heated for 24 h, followed by formation of metal oxide nanoparticles indicated by the formation of a dark red color in the solution. The final material was filtered off, rinsed with methanol and water until colorless washings and subsequently oven-dried overnight at 80 °C. FeNP@SBA-15 exhibited 620 m^2^·g^−1^ of surface area and a pore size of 4.8 nm (5–7 nm iron oxide nanoparticle sizes). Typical Fe^3+^ bands at BE 714 eV (Fe2p_3/2_) and 725 eV (Fe2p_1/2_) were observed by XPS for the synthesized catalyst, with only traces (<1%) of zerovalent Fe.

### 2.2. Preparation of 1,8-Dioxo-Octahydroxanthenes

The model reaction comprised the multicomponent reaction between an aldehyde (5 mmol), dimedone (10 mmol) and FeNP@SBA-15 (0.165 g, 0.5 mol%). In a typical reaction run, the mixture of the three components was heated at 80 °C under continuous stirring for a certain time. Reaction completion was monitored by TLC, after which the mixture was left to cool down at room temperature, followed by dissolution in dichloromethane (50 mL) and rotary evaporation to yield the final xanthene product (upon recrystallization in ethanol). The catalyst was recovered from the mixture via filtration, washed with hot ethyl acetate, oven-dried and reused in subsequent reaction runs. All products are well known and were fully characterized by IR and NMR.

## 3. Results and Discussion

The catalytic performance of nanocatalysts is well known to depend on morphology, particle size and structure of nanoparticles [44]. A number of conventional techniques such as X-ray diffraction (XRD), X-ray photoelectron spectroscopy (XPS), transmission electron microscopy (TEM), scanning electron microscopy (SEM) and inductively coupled plasma/mass spectrometry (ICP/MS) have been used to study textural and morphological properties of FeNP@SBA-15 catalysts [44].

We have previously reported in our earlier papers about the catalytic performance of FeNP@SBA-15 in various types of organic transformations including oxidation of sulfides to sulfoxides [44], esterification of carboxylic acids [45], oxidation of styrene derivatives [46] and oxidative esterification of alcohols and aldehydes (Table S1) [47]. The results of the mentioned reports confirmed the high catalytic activities of supported FeNP in different conditions.

To ascertain the optimum amount of FeNP@SBA-15 to use and select optimum synthetic conditions, a model reaction was selected based on the use of benzaldehyde and dimedone as reagents. As seen in Table 1, entry 1, 3,3,6,6-tetramethyl-9-phenyl-3,4,5,6,7,9-hexahydro-1H-xanthene-1,8(2H)-dione was only obtained in poor yields in the absence of FeNP@SBA-15 at 100 °C or higher temperatures.

According to the experimental results above, the efficiency of the FeNP@SBA-15 was initially found to be influenced by both the amount of the catalyst and the solvent nature. Results under solventless conditions provided improved catalytic performance of FeNP@SBA-15 (Table 1, entries 1, 6–17). By adding a small amount of FeNP@SBA-15 to the model reaction mixture, the rate of reaction was dramatically accelerated under solventless conditions, leading to completion within 30 min (Table 1, entry 16). Under such optimized results, the scope of the reaction was further investigated for the preparation xanthene derivatives using a variety of substituted benzaldehydes.

Table 2 shows that this system can be easily applied to various structurally different benzaldehyde containing electron-releasing or withdrawing group. The results of the optimized reaction in Table 2 shows that rates of reaction can be affected by different substituents in the aromatic rings. It is obvious that electron-withdrawing groups improved both yield and the rate of reaction through the activation of aromatic rings (Table 2, entries 2–4). On the other hand, the presence of electron-donating groups led to slower reaction rates (and reduced yields) as compared to electron-withdrawing groups (Table 2, entries 8 and 9).

The efficiency of FeNP@SBA-15 as catalyst in the proposed synthesis was further compared with a range of literature reported data for the same chemistries (Table 3) [48,49,50,51,52,53,54,55]. Results demonstrated that our method can provide excellent yields at moderate times of reaction with respect to reported procedures.

Furthermore, the stability of the Fe-containing catalyst under the investigated reaction conditions was subsequently explored under optimized conditions. As Table 4 indicates, iron nanoparticles supported on SBA-15 could be recycled and reused twelve times without any appreciable reduction in catalytic activity. No iron leaching was detected in solution (<0.01 ppm, ICP-AES analysis), strongly supporting the stability of the proposed system under the optimized reaction conditions.

**Figure 1:**
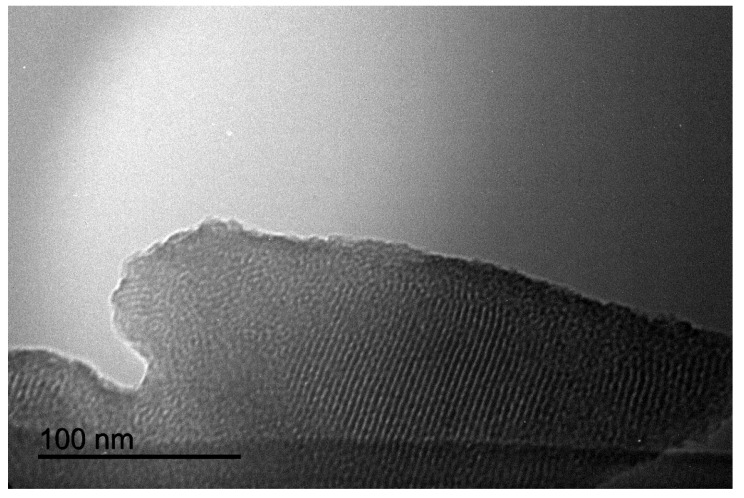
Transmission electron microscopy image of spent FeNP@SBA-15 (after 10 runs).

Figure 1 also depicts a uniform distribution of particle sizes, which can also be observed in the used catalysts, and the high activity of catalysts is preserved well for up to ten runs.

**Scheme 2:**
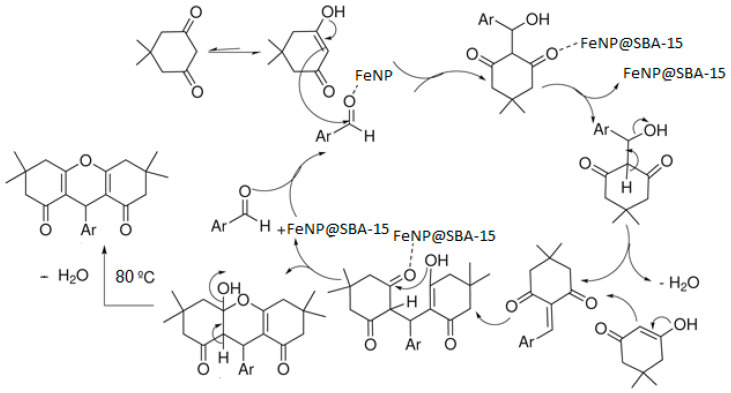
Reaction mechanism for the proposed xanthene syntheses.

The reaction mechanism is shown in Scheme 2 in which the acidity of the Fe-containing material plays a key role in activating the carbonyl group in the first step as well as in the generated intermediate to close the catalytic circle (Scheme 2), generating the xanthene derivatives via final dehydration at 80 °C. A similar reaction mechanism based on similar acid–base carbonyl activation reactions has been recently described for Cirujano et al. using acidic H-USY or Al-MCM supports of metal oxide nanoparticles [56].

## 4. Conclusions

The solventless preparation of 1,8-dioxo-octahydroxanthenes from aromatic aldehydes and dimedone was successfully accomplished employing supported iron oxide nanocatalyst. The proposed catalytic system was found to be highly stable and reusable (up to 12 times), recovered by using simple filtration, without any activity loss. Effectiveness, generality, less reaction time, high yields, low catalyst loading, simplicity and easy work-up procedure as well as the benefits of neat reaction conditions are promising points for the presented methodology.

## Figures and Tables

**Table 1:** Optimization of synthetic conditions for the synthesis of xanthenes ^a^.

Entry	Catalyst (mol%)	Solvent	Temperature (°C)	Time (min)	Yield 3a (%) ^b^
1	-	-	100	60	trace
2	1	EtOH	reflux	60	92
3	1	CH_3_COCH_3_	reflux	60	72
4	1	CH_3_CN	reflux	60	81
5	1	H_2_O	reflux	60	96
6	1	-	100	60	99
7	1	-	90	60	99
8	1	-	80	60	99
9	1	-	70	60	90
10	1	-	80	45	99
11	1	-	80	30	99
12	1	-	80	20	92
13	0.5	-	80	30	99
14	0.3	-	80	30	99
15	0.2	-	80	30	99
16	0.1	-	80	30	99
17	0.08	-	80	30	89

^a^ Reaction conditions: dimedone (2 mmol), benzaldehyde (1 mmol); ^b^ Isolated yields.

**Table 2:** Preparation of 1,8-dioxo-octahydroxanthene^a^ derivatives using Fe@SBA-15 as catalyst.

Entry	Aldehyde	Time (min)	Yield (%) ^a^	MP (°C)	Literature MP	Ref.
1	Benzaldehyde	30	99	204–206	203–205	[47]
2	4-Nitrobenzaldehyde	20	99	218–221	222–224	[50]
3	3-Nitrobenzaldehyde	20	98	169–172	168–170	[31]
4	2-Nitrobenzaldehyde	30	95	203–205	203–205	[47]
5	4-Chlorobenzaldehyde	20	97	235–238	233–235	[47]
6	2,4-Dichlorobenzaldehyde	40	95	253–255	254–255	[35]
7	2-Bromobenzaldehyde	45	90	220–223	221–223	[49]
8	4-Methylbenzaldehyd	55	92	216–218	217–218	[51]
9	4-Methoxybenzaldehyde	60	94	245–246	241–243	[50]
10	2-Chlorobenzaldehyde	45	90	227–230	228–230	[51]
11	4-Hydroxybenzaldehyde	60	90	244–247	245–247	[52]
12	4-Fluorobenzaldehyde	20	97	230–231	235–236	[48]
13	3-Chlorobenzaldehyde	40	95	190–192	190–192	[53]
14	4-Bromobenzaldehyde	20	95	238–240	241–243	[49]

Reaction conditions: dimedone (2 mmol), aldehyde (1 mmol), 0.001 mmol catalyst, 80 °C; ^a^ Isolated yield.

**Table 3:** Comparative performance of FeNP@SBA-15 with literature reported catalytic systems.

Entry	Catalyst	Catalyst Loading (mol%)	T (°C)	Time (min)	Yield (%)	Ref.
1	FeNP@SBA-15	0.1	80	30	99	This study
2	Silica-Supported Preyssler nanoparticles	0.5	Reflux	3 h	93	[30]
3	Nano-TiO_2_	10	100	30	90	[50]
4	[nano-Fe_3_O_4_@SiO_2_@(CH_2_)_3_-Imidazole-SO_3_H]Cl	0.01	80	25	92	[51]
5	Fe_2_(SO_4_)_3_·7H_2_O	10	120	1.5 h	86	[52]
6	p-Toluene Sulfonic Acid	30	80	30	99	[53]
7	CuS quantum dots	0.006 gr	80	6	95	[55]

**Table 4:** Reuses of the supported FeNP catalyst in the reaction of benzaldehyde with dimedone.

Run No. ^a^	1	2	3	4	5	6	7	8	9	10	11	12
Yield (%) ^b^	99	99	99	99	98	98	98	97	97	96	94	92

^a^ Reaction conditions: benzaldehyde (5.0 mmol) and dimedone (10.0 mmol), supported FeNP@SBA-15 (0.005 mmol, 0.167g) at 80 °C for 30 min; ^b^ Isolated yields.

## Supplementary Materials

The following are available online at https://www.mdpi.com/1996-1944/12/15/2386/s1, Table S1: Selected spectroscopic data.
