# Correction: Rajabi et al. Solvent-Free Preparation of 1,8-Dioxo-Octahydroxanthenes Employing Iron Oxide Nanomaterials. *Materials* 2019, *12*, 2386

**DOI:** 10.3390/ma17112766

**Published:** 2024-06-06

**Authors:** Fatemeh Rajabi, Mohammad Abdollahi, Elham Sadat Diarjani, Mikhail G. Osmolowsky, Olga M. Osmolovskaya, Paulette Gómez-López, Alain R. Puente-Santiago, Rafael Luque

**Affiliations:** 1 Department of Science, Payame Noor University, Tehran 19569, Iran; mohammadabdollahichem@gmail.com; 2 Department of Chemistry, University of Guilan, Rasht 1914, Iran; diarjanielham@gmail.com; 3 Institute of Chemistry, Saint-Petersburg State University, Saint-Petersburg 198504, Russia; m_osmolowsky@mail.ru (M.G.O.); o_osmolowskaya@mail.ru (O.M.O.); 4 Department of Organic Chemistry, University of Cordoba, Campus de Rabanales, Edificio Marie Curie (C-3), Ctra Nnal IV-A, Km 396, E14014 Cordoba, Spain; gomezl.paulette@gmail.com (P.G.-L.); apuentesantiago@gmail.com (A.R.P.-S.); q62alsor@uco.es (R.L.); 5 Peoples Friendship University of Russia (RUDN University), 6 Miklukho Maklaya str., 117198 Moscow, Russia

It has been brought to the attention of the Editorial Office that Figure 1 in the original publication [1] contained a duplicated picture that was mistakenly included in the paper. The authors acknowledge that this error occurred due to the similarity in the file names, which went unnoticed during the preparation of the figures. The corrected Figure 1 appears below. The authors state that the scientific conclusions are unaffected. This correction was approved by the Academic Editor. The original publication has also been updated.

## Figures and Tables

**Figure 1:**
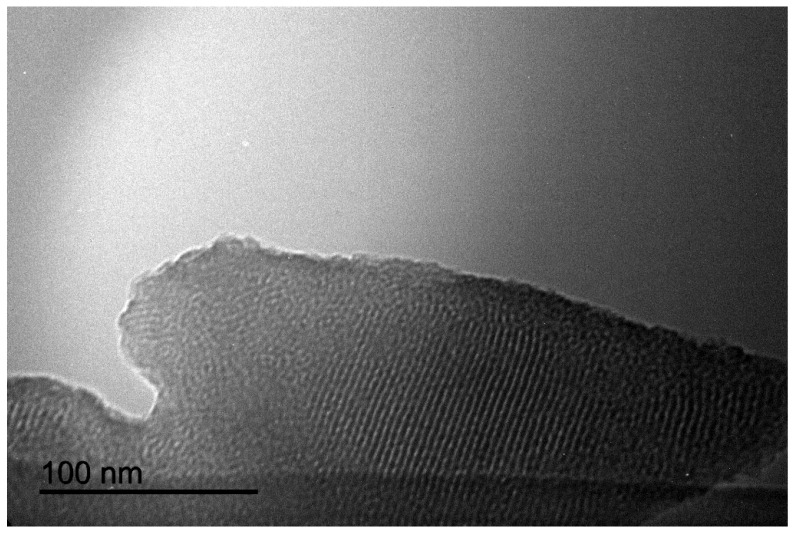
Transmission electron microscopy image of spent FeNP@SBA-15 (after 10 runs).
